# Neonatal Selenium Deficiency Decreases Selenoproteins in the Lung and Impairs Pulmonary Alveolar Development

**DOI:** 10.3390/antiox11122417

**Published:** 2022-12-07

**Authors:** Laura G. Sherlock, William C. McCarthy, Maya R. Grayck, Mack Solar, Andres Hernandez, Lijun Zheng, Cassidy Delaney, Trent E. Tipple, Clyde J. Wright, Eva S. Nozik

**Affiliations:** 1 Perinatal Research Center, Department of Pediatrics, University of Colorado Anschutz Medical Campus, Aurora, CO 80045, USA; 2 Department of Pediatrics, University of Oklahoma College of Medicine, Oklahoma City, OK 73104, USA; 3 Cardiovascular Pulmonary Research Laboratories, Departments of Pediatrics and Medicine, University of Colorado Anschutz Medical Campus, Aurora, CO 80045, USA

**Keywords:** selenium, lung, bronchopulmonary dysplasia, pulmonary development, antenatal nutrition, glutathione peroxidase, thioredoxin reductase, selenoprotein N

## Abstract

Decreased selenium (Se) levels during childhood and infancy are associated with worse respiratory health. Se is biologically active after incorporation into Se-containing antioxidant enzymes (AOE) and proteins. It is unknown how decreased maternal Se during pregnancy and lactation impacts neonatal pulmonary selenoproteins, growth, and lung development. Using a model of neonatal Se deficiency that limits Se intake to the dam during pregnancy and lactation, we evaluated which neonatal pulmonary selenoproteins are decreased in both the saccular (postnatal day 0, P0) and early alveolar (postnatal day 7, P7) stages of lung development. We found that Se deficient (SeD) pups weigh less and exhibit impaired alveolar development compared to Se sufficient (SeS) pups at P7. The activity levels of glutathione peroxidase (GPx) and thioredoxin reductase (Txnrd) were decreased at P0 and P7 in SeD lungs compared to SeS lungs. Protein content of GPx1, GPx3 and Txnrd1 were decreased in SeD lungs at P0 and P7, whereas Txnrd2 content was unaltered compared to SeS controls. The expression of NRF-2 dependent genes and several non-Se containing AOE were similar between SeS and SeD lungs. SeD lungs exhibited a decrease in selenoprotein N, an endoplasmic reticulum protein implicated in alveolar development, at both time points. We conclude that exposure to Se deficiency during pregnancy and lactation impairs weight gain and lung growth in offspring. Our data identify multiple selenoproteins in the neonatal lung that are vulnerable to decreased Se intake, which may impact oxidative stress and cell signaling under physiologic conditions as well as after oxidative stressors.

## 1. Introduction

Poor nutrition during and after pregnancy can negatively impact lung development and pulmonary health in the offspring [1,2,3,4]. While much of this prior research focuses on the roles of macronutrients [1,2] or overall growth [3,4,5], there is accumulating evidence that micronutrients such as selenium (Se) also contribute to lung development [6,7,8]. Se is an essential trace mineral important in regulating the physiologic redox state and response to oxidative challenges [9,10]. Se deficiency is a common micronutrient deficiency, impacting 500 million to 1 billion individuals globally [11]. Low circulating Se levels have been associated with poor respiratory health in infants and children, including an increased incidence of childhood wheeze [12], pediatric asthma [12,13,14], worse pulmonary function in school-aged children [15], and prolonged ventilatory courses in inflamed pediatric patients [16]. Clinical evidence indicates that lung development can be impaired early after birth when a baby is Se deficient. Specifically, Se deficiency in preterm infants is associated with increased oxygen requirement at 28 days and an increased incidence of the chronic lung disease of prematurity, bronchopulmonary dysplasia (BPD) [9,10,11].

There are few preclinical studies exploring how neonatal or juvenile Se deficiency negatively impacts respiratory health. Kim et al. published that Se deficient neonatal rats exhibits greater hyperoxia-induced lung injury when compared to Se sufficient neonatal rats [17]. The authors also demonstrate that Se deficient pups exhibit impaired alveolar development under non-hyperoxic conditions, characterized by increased septal attenuation [17]. However, the mechanisms explaining how Se deficiency impairs neonatal pulmonary development under physiologic conditions or after hyperoxia remain incompletely understood.

An infant’s Se status during infancy is regulated by the mother’s Se stores and intake during pregnancy and lactation [18]. After ingestion, Se becomes biologically active when it is incorporated into Se-containing proteins [19]. There are 24 known selenoproteins in rodents and 25 in humans, with a broad range of functions from the regulation of oxidative stress, calcium transport, inflammation, to thyroid function [19]. A group of these selenoproteins are antioxidant enzymes (AOE) called selenoenzymes. Several selenoenzymes have been identified as important for regulating the pulmonary response to oxidative stressors, including the glutathione peroxidases (GPx) and thioredoxin reductases (Txnrd) [20,21]. Preclinical data demonstrates that Se deficiency decreases GPx and Txnrd activity in the lungs of adult rodents [22,23,24] In addition to enhanced oxygen toxicity [17,25,26], Se deficient rodents demonstrate greater lung injury after influenza infection [27,28,29,30] and paraquat exposure [31,32]. It is speculated that diminished pulmonary selenoenzyme expression contribute to worse outcomes after oxidative challenges. Whether these enzymes are similarly impacted in the neonatal lung is unknown.

There is a strong body of literature establishing a hierarchy to Se utilization in states of Se deficiency, sufficiency, and excess [10,33]. The selenoprotein hierarchy has primarily been established in adult rodents, and is reported to differ by age, organ and cell type [33]. It has not yet been evaluated in the neonatal lung. When Se intake is decreased, Se is preferentially distributed to certain selenoproteins and organs over others. For example, when Se supply is limited, the liver is rapidly depleted in the content of multiple selenoproteins, whereas organs such as the brain and testis are resistant to decreased Se supply [33]. The adult lung is sensitive to decreased Se intake [22,34]. GPx content and activity decrease quickly in the adult Se deficient lung [22,34,35]. In contrast, pulmonary Txnrd content and activity decrease only with longer periods of Se depletion [23,35]. Our lab recently published a murine model of antenatal Se deficiency and characterized aspects of the Se hierarchy in neonatal pups at birth [36]. We found that antenatal Se deficiency decreased GPx activity in the circulation, liver and lungs at birth [36].

Decreased abundance of pulmonary selenoproteins during different stages of lung development may contribute to impaired alveolar growth. Thus, we sought to evaluate which specific selenoproteins and selenoenzymes are diminished in the neonatal Se deficient lung. We extended our model of antenatal Se deficiency to restrict Se intake during both gestation and lactation. We then assessed lung development at both the saccular and alveolar stages. We hypothesized that perinatal Se deficiency will decrease neonatal pulmonary selenoprotein expression during the saccular and alveolar stages of lung development and will be associated with impaired lung development.

## 2. Materials and Methods

### 2.1. Model of Neonatal Se Deficiency

Selenium deficiency was induced in C57Bl/6 breeding pairs as previously described [36]. Briefly, adult male and female mice were allocated to Torula yeast-based Se sufficient (SeS) diet, providing 0.4 ppm sodium selenite, or a Se deficient (SeD) diet, containing <0.01 ppm sodium selenite. These mice were fed SeS or SeD diets for 2–3 weeks prior to the onset of breeding, then were set to breed. Dams were allowed to serially gestate and nurse. After birth, pups were nursed by the dams that gestated them. Pups were euthanized for organ collection at postnatal day 0 (P0) and postnatal day 7 (P7). In this study, 4 SeS dams and 4 SeD dams were used. For the neonatal pups, 21 SeS and 23 SeD pups were used. Pups were sexed visually at time of sacrifice, as described in prior reports from our and other groups [36,37]. Institutional Animal Care and Use Committee (IACUC) at the University of Colorado (Aurora, CO, USA) approved of these procedures. National Institutes of Health guidelines for ethical animal treatment were used for the care and handling of study animals.

### 2.2. Collection of Blood and Organs

Pentobarbital sodium (200 mg/kg) was delivered by intraperitoneal injection. The chest was opened and blood removed from the right ventricle, before the lungs were flushed by perfusing the pulmonary artery with 5 mL of phosphate-buffered saline (PBS). Lungs were then extracted and placed in liquid nitrogen to snap freeze before being stored at −80 degrees Celsius.

### 2.3. Morphometric Analysis

Radial alveolar counts (RAC) and mean linear intercept (MLI) were performed on hematoxylin and eosin stained slides. These morphometric analyses were performed on 7–8 male and female mice from each dietary group. (RAC) were assessed as previously described [38]. First, a terminal bronchiole was identified, next, a line was drawn to the periphery of the lung; then the intersections of lung alveoli were counted. At least 4 RACs were counted for each sample. MLI was measured with the image-analysis program Metamorph Basic, Version 7 (Molecular Devices, Sunnyvale, CA, USA). A computer-assisted, custom-designed macro was used on images that were taken on an Olympus IX83 microscope (10×, 20×, and 40× objective) and quantified with Metamorph Basic (Molecular Devices, Sunnyvale, CA, USA). At least 8 non-overlapping sections per mouse were assessed at 20× magnification. Fields with large airways or vessels were excluded. The analysis was performed by an investigator blinded to the experiment group.

### 2.4. Glutathione Peroxidase Activity

Pulmonary glutathione peroxidase activities were determined as previously described [39]. Briefly, 20 miligram (mg) of lung was lysed in 200 microliters (uL) of 50 mM Tris/Hcl with 5 mM EDTA and 1 mM sodium azide (to block endogenous catalase), and additionally Halt protease and phosphatase inhibitors (ThermoFisher, Waltham, MA, USA) (1:100). These lysates spun at 14,000 G for 10 min, after which the supernatant was removed. Protein content was determined by Bradford. 35 micrograms (ug) of sample was then incubated with a reaction buffer for 10 min at 37 degrees Celsius. To initiate the reaction, hydrogen peroxide was added to the sample-reaction mix, with a final concentration of 50 μM. This was then monitored with a microplate reader for a duration of 2 min at 340 nm. Glutathione peroxidase activity was calculated using Lambert-Beer’s law, with 1 U of activity defined as the consumption of 1 μmol nicotinamide adenine dinucleotide phosphate (NADPH)/min/mL.

### 2.5. Thioredoxin Reductase (Txnrd) Activity Level

The activity level of thioredoxin reductase was determined indirectly with an insulin disulfide reduction assay with NADPH and dithio-bis-(2-nitrobenzoic acid) (DTNB), as previously described and using manufacturer instructions (Cayman Chemicals, Ann Arbor, MI, USA) [40,41,42].

### 2.6. Immunoblot Analysis

Thirty to forty ug of protein was electrophoresed on a 4–12% polyacrylamide gel (Invitrogen, Waltham, MA, USA). Proteins were transferred to an Immobilon membrane (Millipore, Burlington, MA, USA). Total protein was determined using Revert Total Protein Stain (LiCor, Lincoln, NE, USA), then membranes were blocked in 2% milk for one hour at room temperature before primary antibodies added (See Supplementary Table S1 for antibodies and dilutions). Secondary antibodies were in the appropriate host (1:5000). Blots were imaged utilizing the LiCor Odyssey system. Densitometry analysis was performed by measuring an equal sized area of the bands of the predicted molecular weight for each antibody using ImageStudio Version 4 (LiCor, Lincoln, NE, USA).

### 2.7. Analysis of Relative mRNA Levels by RT-qPCR

Frozen tissue was placed in RLT buffer (Qiagen) and homogenized using the Bullet Blender (NextAdvance, Troy, NY, USA). Pulmonary mRNA was isolated using RNeasy Mini Kit (Qiagen, Germantown, MD, USA). RNA was assessed for quality and concentration by Nanodrop (ThermoFisher Scientific, Waltham, MA, USA). cDNA was synthesized at 1 μg/20 μL using Verso cDNA synthesis kit (ThermoFisher Scientific, Waltham, MA, USA). Relative mRNA levels were evaluated by quantitative real-time PCR using exon spanning primers, Taqman gene technology and StepOnePlus Real-Time PCR (Applied Biosystems, Foster City, CA, USA) (See Supplementary Table S2 for primers). Relative quantification was performed with the cycle threshold method (ΔΔCT) normalizing to 18S.

### 2.8. Statistical Analysis

Comparisons were made between dietary exposure groups, and the null hypothesis that no difference existed between and within groups tested by Student’s two-sided *t*-tests for experiments. When data were evaluated for sex differences and dietary exposure, comparisons were made by two-way ANOVA with Tukey’s method for multiple comparisons. The males and females were grouped together for analyses and the analyses were repeated to evaluate for sex-based differences. Statistical significance between groups was defined at *p* < 0.05 and were analyzed using Prism (Version 9.3.1, Graphpad Software, San Diego, CA, USA).

## 3. Results

### 3.1. Neonatal Se Deficiency Impairs Postnatal Growth at Day of Life 7 through Adulthood

**Figure 1:**
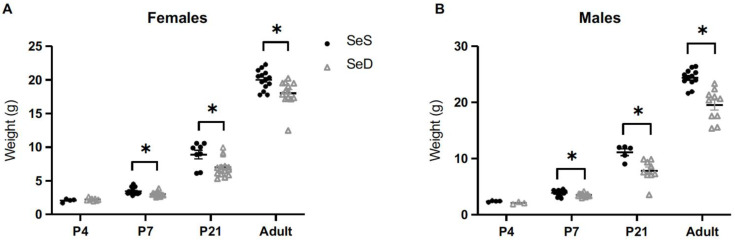
Neonatal Se deficient mice exhibit lower weights than Se sufficient mice from postnatal day 7 through adulthood. C57Bl/6 mice were placed on diets that differed only in Se content, either 0.4 ppm or <0.01 ppm of sodium selenite, through pregnancy and lactation. After weaning, pups were continued on their respective SeS or SeD diets. Female and male offspring were weighed at postnatal day 0, 4, 7, 21 and 8–10 weeks of life. (**A**) Weights for female mice (**B**) Weights for male mice N = 4–15 for all groups. Data are presented as mean (±SEM), * *p* < 0.05 vs. age-matched SeS control by Students two-sided *t*-test.

Selenium deficiency is associated with several markers of poor health in infancy, including postnatal growth failure [43]. We have previously reported that pups born to Se deficient dams have similar weights at birth in comparison to pups born to Se sufficient dams [36]. To evaluate the effect of continued Se deficiency during lactation on neonatal somatic growth, we assessed weights of Se sufficient (SeS) and Se deficient (SeD) pups at postnatal days 4, 7, and 21, and 8–10 weeks of life. We found that SeD female and male pups weighed less than SeS female and male pups at postnatal days 7 and 21, and 8–10 weeks (Figure 1A,B).

### 3.2. Neonatal Se Deficiency Impairs Alveolar Development

**Figure 2:**
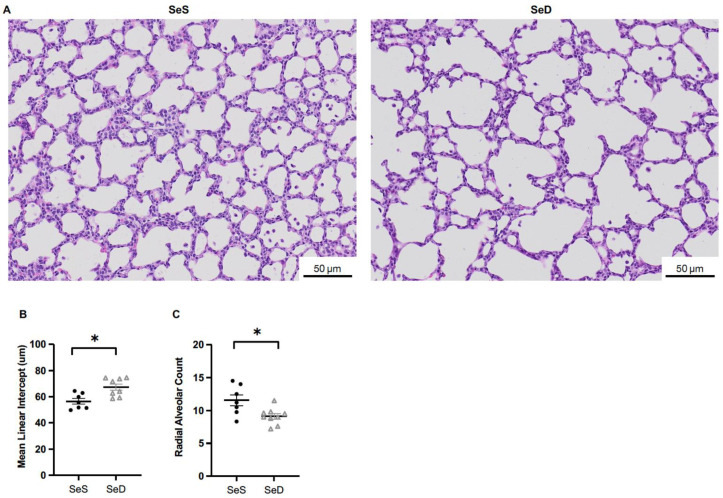
Neonatal Se deficient mice demonstrate impaired alveolar development at postnatal day 7. C57Bl/6 mice were placed on diets that differed only in Se content, either 0.4 ppm or <0.01 ppm of sodium selenite, through pregnancy and lactation. Alveolar development was assessed at postnatal day 7. (**A**) Representative images of lungs, (**B**) Mean linear intercept, (**C**) Radial alveolar counts. N = 7–9 for all groups. Data are presented as mean (±SEM), * *p* < 0.05 vs. SeS control by Students two-sided *t*-test.

Neonatal Se deficient rats demonstrate impairments in alveolar growth [17]. We sought to determine if alveolar growth was similarly impaired in neonatal Se deficient mice at postnatal day 7, an early time point in the alveolar stage of lung development. We selected this time point as it is the stage of murine development consistent with a human neonate born at term. We measured mean linear intercept (MLI), an objective measurement of alveolar development that reflects the average distance between alveoli [44,45]. MLI was increased in Se deficient pups compared to Se sufficient pups (Figure 2A,B). We next measured RAC, another objective measurement of alveolar development that assesses the number of alveoli [46,47]. RAC were lower in Se deficient pups when compared to Se sufficient pups (Figure 2A,C). Sex has been reported as a significant biological variable in neonatal hyperoxia studies, where male mice are more susceptible to injury than females [48]. Thus, we evaluated our results by sex and found no differences in either MLI or RAC between SeS or SeD male and female pups (Supplemental Figure S1).

### 3.3. Neonatal Se Deficiency Decreases Glutathione Peroxidase and Thioredoxin Reductase Activity in the Lung

**Figure 3:**
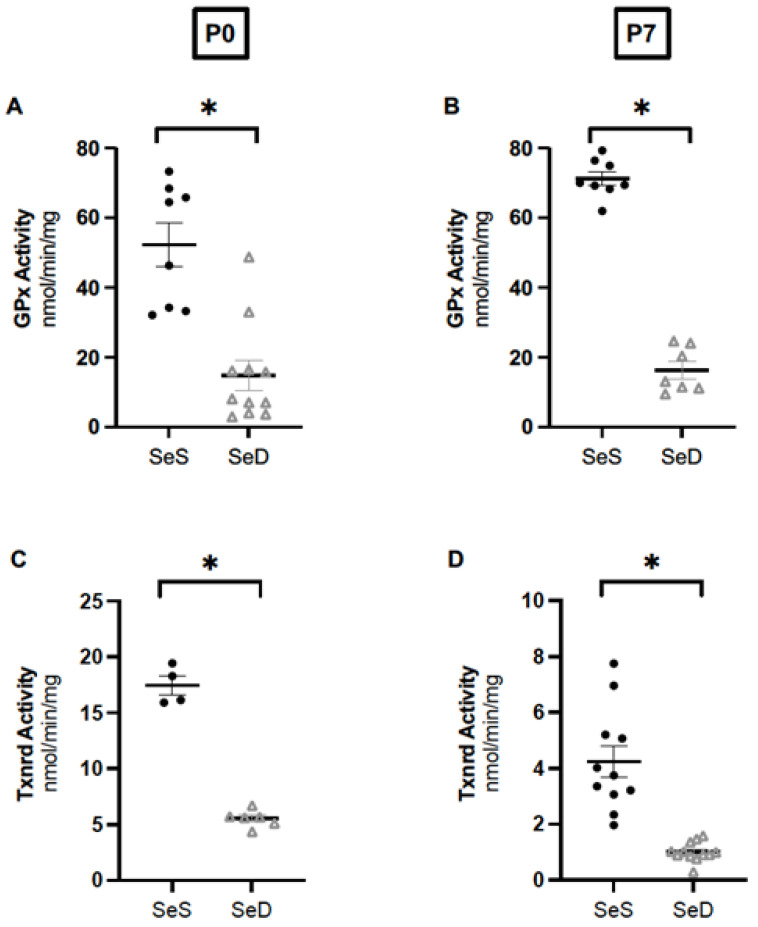
Neonatal Se deficient mice demonstrate decreased activity of GPx and Txnrd at postnatal day 0 and 7. C57Bl/6 mice were placed on diets that differed only in Se content, either 0.4 ppm or <0.01 ppm of sodium selenite, through pregnancy and lactation. Pulmonary organ homogenate was evaluated on day of birth (P0) and postnatal day 7 (P7). (**A**) Glutathione peroxidase activity levels at P0, (nmol/minute/mg of protein) (**B**) Glutathione peroxidase activity level at P7, (nmol/minute/mg of protein). (**C**) Thioredoxin reductase activity levels at P0, (nmol/minute/mg of protein). (**D**) Thioredoxin reductase activity level at P7, (nmol/minute/mg of protein). N = 4–12 for all groups. Data are presented as mean (±SEM), * *p* < 0.05 vs. SeS control by Students two-sided *t*-test.

After observing impaired alveolar development, we sought to determine if neonatal Se deficiency decreased the activity of two Se-containing antioxidant enzymes (AOE) demonstrated to decrease in adult Se deficient lungs, GPx and Txnrd2. We have previously reported that pups born after antenatal Se deficiency exhibit decreased GPx activity in the lung at birth [36]. We confirmed our prior results and found that neonatal Se deficiency decreased GPx activities in the lung at both P0 and P7 (Figure 3A,B). Next, we measured Txnrd and found that neonatal Se deficiency also decreased Txnrd activity in SeD lungs at both P0 and P7 (Figure 3C,D). Studies in adult mice reveal sex dimorphisms for the activity of some selenoenzymes, including GPx [33]. Our prior report in neonatal mice found no sex differences in circulating or hepatic GPx activity between males and female Se sufficient and Se deficient pups at birth, but it is unknown when sex differences emerge [36]. Thus, we evaluated for sex differences and found that GPx and Txnrd activity in the lungs of Se sufficient male and female pups were not different from one another at P0 or P7 (Supplemental Figure S2). We also found that GPx and Txnrd activities in the lungs of Se deficient male and female pups were equivalent between one another at both ages (Supplemental Figure S2).

### 3.4. Neonatal Se Deficiency Decreases Pulmonary GPx1, GPx3 and Txnrd1 Content; Txnrd2 Content Is Preserved

**Figure 4:**
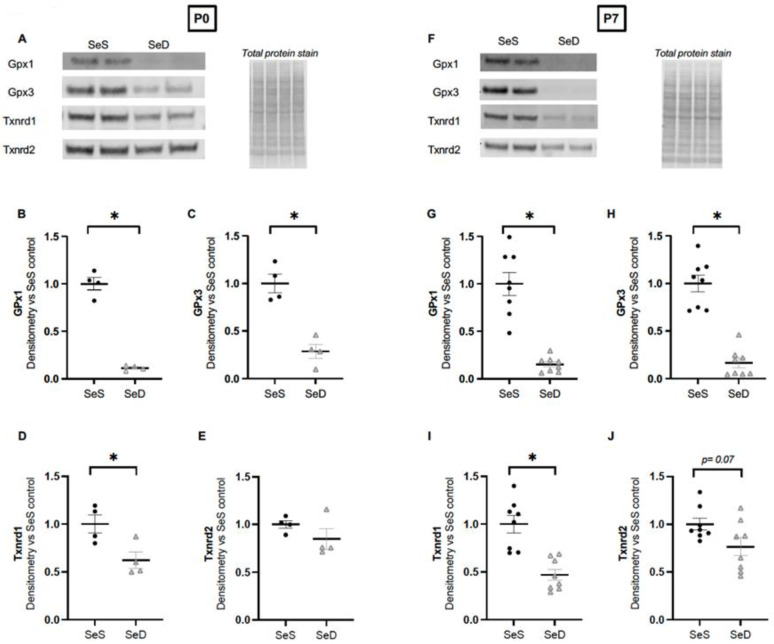
Se deficient neonates demonstrate decreased pulmonary GPx1, GPx3, and Txnrd1 content; Txnrd2 content is preserved. C57Bl/6 mice were placed on diets that differed only in Se content, either 0.4 ppm or <0.01 ppm of sodium selenite, through pregnancy and lactation. Pulmonary organ homogenate was evaluated on day of birth (P0) and postnatal day 7 (P7). (**A**) Representative Western blots of pulmonary Gpx1, Gpx3, Txnrd1 and Txnrd2 for SeS P0 samples and SeD P0 samples, Densitometric analysis of (**B**) Gpx1, (**C**) Gpx3, (**D**) Txnrd1, and (**E**) Txnrd2 protein content at P0. (**F**) Representative Western blots of pulmonary Gpx1, Gpx3, Txnrd1 and Txnrd2 for SeS P7 samples and SeD P7 samples, Densitometric analysis of (**G**) Gpx1, (**H**) Gpx3, (**I**) Txnrd1, and (**J**) Txnrd2 protein content expression at P7. Results are normalized to total protein stain and expressed as a ratio to SeS mice. N = 4–8 for all groups. Data and presented as mean (±SEM), * *p* < 0.05 vs. SeS age matched control, by Students two-sided *t*-test.

A complex hierarchy Se-dependent protein tissue-specific expression is well described in adult rodents, wherein quantities of specific selenoproteins decrease rapidly in the setting of limited Se supply while others are preferentially preserved [49]. This is reported to differ by age, organ and cell type, and has not been evaluated in the neonatal lung [33]. Elucidation of the neonatal lung selenoprotein hierarchy in the setting of Se deficiency may provide insights into susceptibility to different exposures. Thus, we measured the protein content of several key GPx and Txnrd isoforms in neonatal lungs. GPx1 and GPx3 have both been reported to as important to the pulmonary response to oxidative stressors in adult rodent, thus we first measured the content of GPx1 and GPx3 in the neonatal lung [20,21,50]. We found that both GPx1 and Gpx3 were significantly lower in SeD lungs at both P0 and P7 when compared to SeS lungs (Figure 4A–C,F–H). Embryonic deletion of Txnrd1 or Txnrd2 lethal, suggesting an essential role in embryogenesis and organ development [51,52]. Our data revealed that Txnrd1 content was significantly decreased in P0 and P7 SeD lungs (Figure 4A,D,F,I). In contrast, Txnrd2 content was not significantly different between SeS and SeD lungs at either time point (Figure 4A,E,F,J).

### 3.5. Neonatal Se Deficiency Decreases Transcription of Pulmonary Glutathione Peroxidase 1 and 3

**Figure 5:**
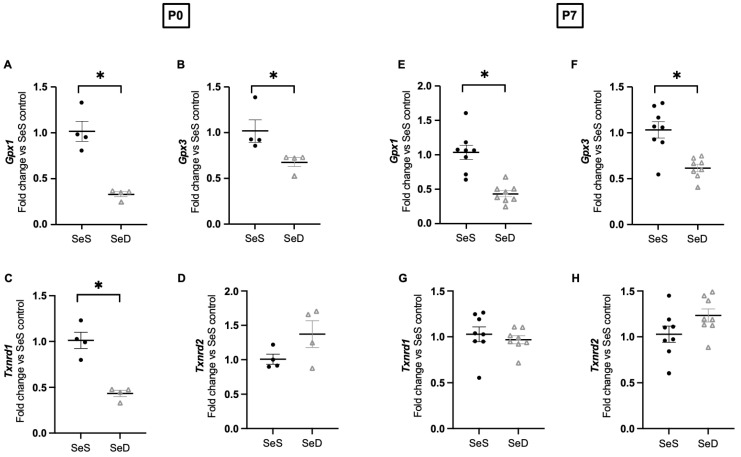
Se deficient lungs exhibit decreased *Gpx1* and *Gpx3* gene transcription at P0 and P7. C57Bl/6 mice were placed on diets that differed only in Se content, either 0.4 ppm or <0.01 ppm of sodium selenite, through pregnancy and lactation. Pulmonary organ homogenate was evaluated on day of birth (P0) and postnatal day 7 (P7). Fold change (**A**) *Gpx1*, (**B**) *Gpx3,* (**C**) *Txnrd1*, and (**D**) *Txnrd2* (normalized to SeS) at P0. Fold change (**E**) *Gpx1*, (**F**) *Gpx3,* (**G**) *Txnrd1*, and (**H**) *Txnrd2* are (normalized to SeS) at P7. N = 4–8 for all groups. Data presented as mean (±SEM), * *p* < 0.05 vs. SeS age matched control, by Students two-sided *t*-test.

Decreased selenoprotein expression during limited Se conditions can occur due to decreased transcription and/or post-translational modifications [19]. To evaluate the impact of Se deficiency on pulmonary selenoenzymes, we measured the mRNA transcripts of *Gpx1*, *Gpx3*, *Txnrd1* and *Txnrd2* at P0 and P7. At P0, we found that SeD lungs exhibited decreased mRNA transcripts for *GPx1*, *Gpx3* and *Txnrd1* (Figure 5A–C), and similar *Txnrd2* mRNA transcripts compared to SeS lungs (Figure 5D). At P7, we also observed decreased mRNA transcripts for *GPx1* and *GPx3* in SeD lungs (Figure 5E,F). Transcript levels or *Txnrd1* and *Txnrd2* were inbetween SeS and SeD pups (Figure 5G,H).

### 3.6. Neonatal Se Deficiency Does Not Alter Transcription of NRF-2 Targets in the Lung

**Figure 6:**
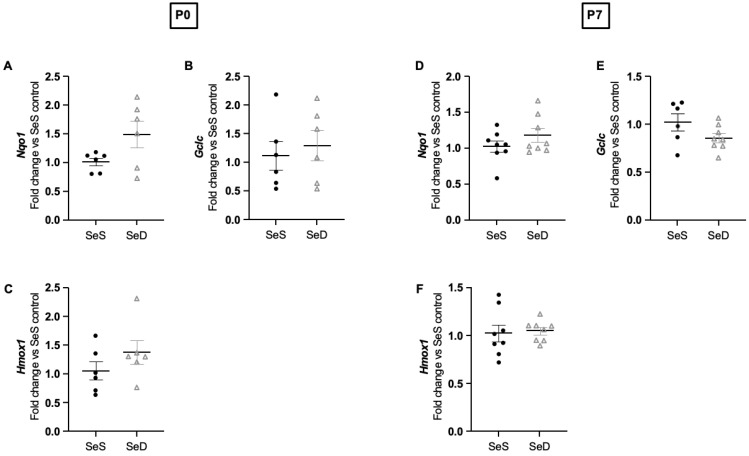
Se deficient neonates have unaltered pulmonary NRF-2 dependent gene transcription at P0 and P7. C57Bl/6 mice were placed on diets that differed only in Se content, either 0.4 ppm or <0.01 ppm of sodium selenite, through pregnancy and lactation. Pulmonary organ homogenate was evaluated on day of birth (P0) and postnatal day 7 (P7). Fold change (**A**) *Nqo1*, (**B**) *Gclc*, (**C**) *Hmox1* (normalized to SeS) at P0. Fold change in (**D**) *Nqo1*, (**E**) *Gclc*, (**F**) *Hmox1* (normalized to SeS) at P7. N = 4–8 for all groups. Data and presented as mean (±SEM).

Se deficiency induces Nuclear factor erythroid 2-related factor 2 (NRF2) dependent gene transcription in adult rodents [53]. Pharmacologic inhibition of Txnrd similarly increases NRF2-regulated gene transcription in the lungs of neonatal mice [54]. To evaluate the impact of dietary Se deficiency on NRF-2 related gene transcription in the neonatal lung, we measured the mRNA transcripts for four NRF-2 regulated targets: nicotinamide adenine dinucleotide phosphate reduced quinone oxidoreductase-1 (*Nqo1*), glutamate-cysteine ligase (*Gclc*), and heme oxygenase 1 (*Hmox1*). At P0, we found no differences in the mRNA levels between SeS and SeD lungs (Figure 6A–C). Similarly, at P7, we found no differences in transcript levels between SeS and SeD lungs (Figure 6D–F).

### 3.7. Neonatal Se Deficiency Does Not Increase Pulmonary Content of Non-Se Containing Antioxidant Enzymes at P0 or P7

**Figure 7:**
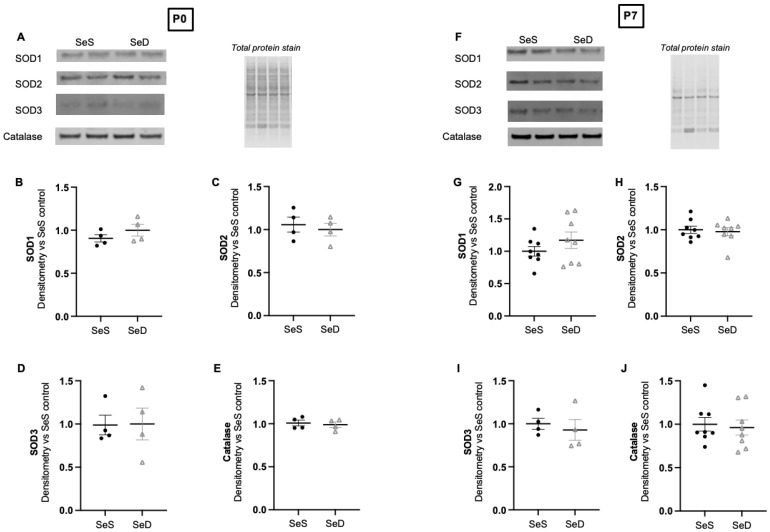
Se deficient neonates have unaltered protein content of the SODs and catalase at P0 and P7 C57Bl/6 mice were placed on diets that differed only in Se content, either 0.4 ppm or <0.01 ppm of sodium selenite, through pregnancy and lactation. Pulmonary organ homogenate was evaluated on day of birth (P0) and postnatal day 7 (P7). (**A**) Representative Western blots of pulmonary SOD1, SOD2, SOD3 and catalase for SeS P0 samples and SeD P0 samples, Densitometric analysis of (**B**) SOD1, (**C**) SOD2, (**D**) SOD3, and (**E**) Catalase protein content expression at P0. (**F**) Representative Western blots of pulmonary SOD1, SOD2, SOD3 and catalase for SeS P7 samples and SeD P7 samples, Densitometric analysis of (**G**) SOD1, (**H**) SOD2, (**I**) SOD3, and (**J**) Catalase protein content expression at P7. Results are normalized to total protein stain and expressed as a ratio to SeS mice. N = 4–8 for all groups. Data and presented as mean (±SEM).

Se deficiency is demonstrated to increase several non-Se containing antioxidant enzymes (AOE) in the adult liver, including superoxide dismutase (SOD) [35,55,56]. We have reported that neonatal Se deficiency results in increased SOD2 protein content when Txnrd content is decreased. Thus, to evaluate if neonatal Se deficiency results in compensatory increases in several non-Se containing AOE known to increase after Se deficiency, we first measured the protein content for superoxide dismutase 1 (SOD1), mitochondrial superoxide dismutase 2 (SOD2), and extracellular superoxide dismutase 3 (SOD3). We observed no difference in the protein content of these at either postnatal day 0 or 7 (Figure 7A–D,F–I). As we found a significant decrease in GPx1, a mainly cytoplasmic enzyme that reduces hydrogen peroxide, we next measured the protein content of catalase, another cytoplasmic enzyme that also reduces hydrogen peroxide. We found unaltered catalase content between Se sufficient and deficient lungs at both P0 and P7 (Figure 7A,E,F,J).

### 3.8. Neonatal Se Deficiency Decreases Selenoprotein N, an Endoplasmic Reticulum Selenoprotein Implicated in Normal Alveolar Development, at Both P0 and P7

**Figure 8:**
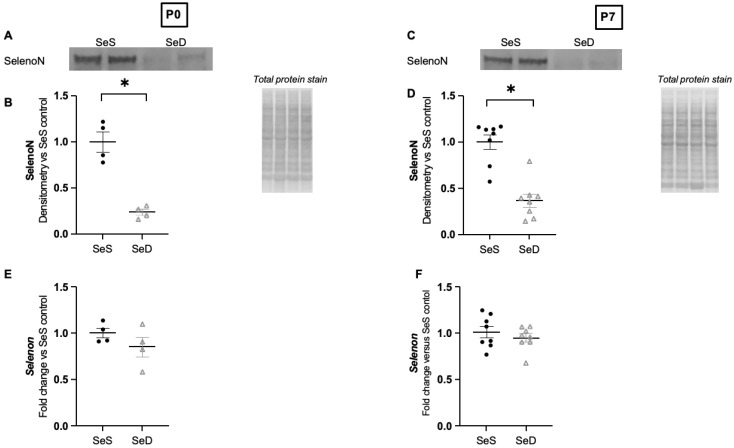
Se deficient neonates demonstrate decreased pulmonary selenoprotein N content at P0 and P7. C57Bl/6 mice were placed on diets that differed only in Se content, either 0.4 ppm or <0.01 ppm of sodium selenite, through pregnancy and lactation. Pulmonary organ homogenate was evaluated for the offspring on day of birth (P0) and postnatal day 7 (P7). (**A**) Representative Western blots of pulmonary selenoprotein N for SeS P0 samples and SeD P0 samples, Densitometric analysis of (**B**) selenoprotein N protein content expression at P0. Results are normalized to total protein stain and expressed as a ratio to SeS mice. (**C**) Fold change in *Selenon* mRNA at P0. (**D**) Representative Western blots of pulmonary selenoprotein N for SeS P7 samples and SeD P7 samples, Densitometric analysis of (**E**) selenoprotein N protein content expression at P7. Results are normalized to total protein stain and expressed as a ratio to SeS mice. (**F**) Fold change in *Selenon* mRNA at P0. N = 4–8 for all groups. Data and presented as mean (±SEM), * *p* < 0.05 vs. SeS age matched control, by Students two-sided *t*-test.

In addition to its importance in AOE function, Se is incorporated into several non-AOE that can impact organ development, including selenoprotein N [19]. Selenoprotein N is an endoplasmic reticulum bound selenoprotein important in myogenesis [57]. Intriguingly, total body selenoprotein N genetic knock-out mice develop impaired alveolar growth [58]. We have not found any reports testing if the protein expression of this selenoprotein is sensitive to Se supply in neonatal or adult mice. Thus, we sought to determine if limited Se supply during pregnancy and lactation decreased selenoprotein N in the lungs of neonatal mice. We found that SeD pups exhibited significantly decreased pulmonary selenoprotein N content at both P0 and P7 (Figure 8A–D). To evaluate if this decrease was transcriptionally regulated, we analyzed the mRNA transcripts for *Selenon* and found no difference in *Selenon* between Se sufficient and deficient pups at P0 or P7 (Figure 8E,F). Selenotranscriptomic analysis demonstrates that sex and organ specific differences in *Selenon* transcription after Se deficiency and excess can occur. Thus, we evaluated for sex differences in selenoprotein N protein expression and mRNA at P7 and did not find a difference between Se sufficient males and females, or Se deficient males and females (Supplemental Figure S3). These results indicate that pulmonary selenoprotein N is regulated by Se supply in infancy and decrease in the Se deficient lung during the saccular and early alveolar stages of lung development.

## 4. Discussion

Se deficiency results in health consequences across the age span and is increasingly recognized as detrimental during fetal and newborn life [9]. Clinical and preclinical work implicates Se deficiency to have negative consequences on pulmonary health, however there are limited prior reports evaluating the impact of Se deficiency on the developing lung [16,17,18,20]. To evaluate the impact of Se deficiency on neonatal lung development and selenoprotein expression, we limited Se supply during both pregnancy and lactation. We evaluated alveolar development and confirmed prior observations in neonatal rats that neonatal Se deficiency results in impaired alveolar growth, with increased MLI and decreased RAC on histologic analysis. We found that body weights of pups exposed to Se deficiency were lower than in Se sufficient pups, starting at postnatal day 7 and continuing through adulthood. To determine the identities of neonatal pulmonary selenoenzymes impacted by limited Se supply, we measured the activities of GPx and Txnrd, and found that Se deficiency decreased activities of both in the saccular and early alveolar stages of lung development. To understand specifically which selenoenzymes were decreased, we measured the protein content of 2 GPx isoforms and 2 Txnrd isoforms, and found that GPx1, GPx3 and Txnrd1 were decreased by neonatal Se deficiency whereas Txnrd2 was unaltered. Se deficiency is demonstrated to increase compensatory redox mechanisms in adult models and the neonatal liver. Thus, we next measured the transcription of NRF-2 dependent genes and the expression of several non-Se-containing AOE, and found that these were similar between SeS and SeD lungs. Finally, to evaluate a non-AOE selenoprotein that has been previously implicated in alveolar development, we measured the protein content of selenoprotein N. We found that selenoprotein N levels were significantly decreased in the neonatal lung by Se deficiency.

Our first novel observation is that that Se deficiency during pregnancy and lactation impairs alveolar growth in neonatal offspring. Histologic evaluation of neonatal Se deficient lungs during the early alveolar stage of lung development revealed increased MLI and decreased RAC compared to Se sufficient lungs. This finding is consistent with a prior study of neonatal rats demonstrating septal attenuation in Se deficient offspring [17]. We expanded upon this prior investigation by evaluating for sex differences and found that the lungs of Se deficient males and females were similarly impacted. Clinically, Se deficiency in preterm infants is associated with an increased risk for bronchopulmonary dysplasia, a disease of impaired alveolar growth [6,59]. Our report adds to the literature implicating neonatal Se status as important for alveolar growth. Presently, it is unknown if these impairments persist into adulthood or correlate with worse pulmonary mechanism, and these will be important questions for future work. While the specific pulmonary cell signaling pathways and cell types impacted by neonatal Se deficiency have not yet been identified, it has been shown that bronchial epithelial cells grown in Se deficient media develop altered cell morphology [29]. Additionally, Se deficient pulmonary microvascular endothelial cells exhibit increased endothelin-1 expression, which is increased in preterm infants with bronchopulmonary dysplasia and pulmonary hypertension [60]. Given the dynamic intercellular communication required for lung development, evaluating the cell specific impact of Se deficiency on alveolar type 2 cells, endothelial cells and myofibroblasts is warranted.

Se deficient pups also demonstrated impaired weight gain compared to Se sufficient counterparts, starting at postnatal day 7 and continuing into adulthood. This finding is consistent with preclinical reports that Se deficiency decreases weight gain in adult and neonatal rodents, and with clinical reports that Se deficiency in infancy is associated with growth stunting [61,62,63,64]. Both fetal and postnatal growth restriction are associated with worse lung development in the neonatal period extending into school age [5,65,66]. The mechanism of postnatal growth failure in the SeD pups is currently unknown. It is possible that hypoalveolarization and impaired pulmonary gas exchange results in suboptimal tissue oxygen delivery, leading to poor growth; or it is possible that diminished Se supply to other organs results in systemic effects that subsequently impair alveolar development. A prior study done in neonatal rats demonstrated that Se deficiency during pregnancy and lactation impaired body weight and linear growth of offspring at weaning, despite the offspring consuming a similar amount of milk per day [38]. In this report, the offspring demonstrated smaller livers, decreased protein content in the liver, and increased hepatic oxidative stress [64]. As the liver is a critical organ for orchestrating postnatal growth, we speculate the hepatic effect of Se deficiency may be a contributing factor to the poor growth in our mice. Children with Se deficiency have been reported to have decreased circulating insulin-like growth factor 1 (IGF-1) [67]. IGF-1 is produced in the liver and is a growth related pathway known to impact pulmonary development [67]. However, it is currently not clear whether hypoalveolarization and poor oxygen delivery or a systemic effect of Se deficiency (such as an impact on IGF-1) explains the growth defect in our study. This could be clarifying in future studies by using lung specific genetic knock-out mice for the selenoprotein tRNA, *Trsp* [68]. Future investigation of how Se deficiency impacts the developing lung will need to evaluate paracrine factors such as IGF-1, in addition to altered local signaling in the lung.

These experiments represent the first evaluation of the neonatal pulmonary selenoprotein hierarchy and establish that multiple neonatal pulmonary selenoenzymes are sensitive to decreased Se supply during gestation and lactation. We found that GPx activity and two of the Se-dependent GPx isoforms (GPx1 and GPx3) decreased in the lungs of Se deficient pups during the saccular and early alveolar stage of lung development. This is consistent with prior reports establishing GPx as highly sensitive to Se intake, including in the lungs of adult rodents [24,34,69]. We also found that Txnrd activity and Txnrd1 protein content were decreased in the Se deficient neonatal lung during the saccular and early alveolar stage. In contrast, Txnrd2 protein content is preserved in Se deficient lungs at both time points. Txnrrd2 knock-out mice are embryonically lethal, indicating there may be an important role for this enzyme during embryogenesis [51]. Txnrd2 is a mitochondrial selenoenzyme, and we speculate that preservation of this enzyme may be important given the role of mitochondrial biology in neonatal pulmonary disease. Insufficient pulmonary selenoenzymes may uniquely predispose the Se deficient infant to increased oxidative stress in the lung. This may carry important clinical implications, as Se deficiency is more prevalent in extremely low gestational age newborns, a population exposed to multiple pulmonary oxidative stressors, including increased oxygen therapy, tracheitis, upper and lower respiratory viral infections, and mechanical ventilation.

We observed no differences in the expression of several NRF-2 regulated genes or the protein contents of superoxide dismutases and catalase between neonatal Se sufficient and deficient lungs at P7. This is in contrast to prior reports demonstrating a compensatory increase in the NRF-2 associated genes *Nqo1*, *Gclc* and *Hmox1*, as well as other reports demonstrating an increase in non-Se containing AOE in lung or liver after dietary Se deficiency as well as genetic or pharmacologic Txnrd inhibition [35,40,41,42,53,54,70,71,72,73,74] There are several possible explanations why the neonatal SeD lung did not elicit a similar response despite decreased Txnrd activity. Differential pulmonary NRF-2 induction has been reported depending on the genetic strain of mouse evaluated, as well as the age and duration of insult [40,54,75]. Specifically, pharmacologic Txnrd inhibition results in a pulmonary NRF-2 induction in C3H/HeN mice, but does not result in a sustained NRF-2 induction in neonatal C57Bl/6 mice [40,54,75] It is possible there are strain dependent or dynamic time related interactions at play, and that we did not capture an early or late adaption. Our next steps include a more comprehensive evaluation of Se deficiency NRF-2 pathways, including testing for pulmonary NRF-2 activation at the mRNA and protein level across the stages of pulmonary development.

Finally, these experiments are the first to demonstrate that pulmonary selenoprotein N is regulated by dietary Se supply. Selenoprotein N is an endoplasmic reticulum bound protein that regulates endoplasmic reticulum calcium uptake [57]. Selenoprotein N is reported to have a high expression in fetal tissues and lower expression in adult tissues [76]. Intriguingly, selenoprotein N knock-out mice demonstrate impaired alveolar growth when evaluated in the alveolar stage of lung development as well as in adulthood [58]. LungMAP indicates that myofibroblasts and endothelial cells express selenoprotein N at high levels during the saccular and early alveolar stage of lung development [77]. As Selenoprotein N knock-out mice demonstrate increased apoptosis in the lung, evaluating for increased apoptosis in the neonatal Se deficient lung and complimentary cell culture will be important future experiments [58].

There are several limitations to our study. Our model induces a severe Se deficiency, by restricting the breeding dams to <0.1 ppm Se during pregnancy and lactation and allowing up to 4 pregnancies on this diet. This model is useful for evaluating how Se deficiency predisposes the neonatal lung to worse outcomes. There are several populations of infants that are at risk for developing Se deficiency, including infants born preterm, infants born in nutritionally Se-poor geographic regions, as well as infants born after antenatal conditions known to negatively impact maternal Se status, such as preeclampsia, gestational diabetes, and chronic infections [78,79]. Clinically, it is thus likely that infants exhibit a range in the degree of Se deficiency. It is not known how short-term or mild Se deficiency would impact pulmonary selenoenzyme expression or lung development at this time. Adult rodent studies of short term Se deficiency have demonstrated worse lung injury after hyperoxia even when pulmonary GPx activity was only 30% lower than Se sufficient control [34]. Including an experimental arm of short term or mild neonatal Se deficiency may be a clinically relevant option for future studies. At this time, we have not assessed if the hypoalveolarization seen at P7 persists into adulthood or impacts pulmonary function. Evaluating pulmonary morphometrics and mechanics in adult mice exposed to Se deficiency as neonates would enhance the clinical implications of our observations. Finally, we have not yet evaluated if restoring pulmonary selenoproteins with postnatal Se supplementation attenuates or prevents altered pulmonary morphometrics. In adult rodents, supplementation with dietary or intraperitoneally delivered Se decreases pulmonary markers of oxidative stress and improves acute lung injury scores after multiple oxidative and inflammatory stressors, including cecal ligation puncture, porcine circovirus type 2 infection, radiation exposure, exhaust inhalation, and tacrolimus, paraquat and aluminum chloride exposure [80,81,82,83,84,85,86]. Future studies will explore the potential of postnatal Se repletion on impaired alveolar development in our novel animal models.

## 5. Conclusions

The neonatal lung is highly susceptible to decreased Se supply during pregnancy and lactation. Se deficiency results in impaired alveolar development in neonatal mice under control conditions. Our study identifies multiple selenoproteins that are diminished in the neonatal Se deficient lung during the saccular and early alveolar stage of lung development. Our studies confirm that nutritional Se deficiency likely alters key cellular signaling pathways required for normal pulmonary growth. We speculate that Se deficiency enhances the susceptibility of the neonatal lung to noxious insults leading to worse pulmonary outcomes.

## Data Availability

Not applicable.

## Supplementary Materials

The following supporting information can be downloaded at: https://www.mdpi.com/article/10.3390/antiox11122417/s1, Table S1: Antibodies used for Western blots analysis; Table S2: Primers used for qPCR analysis; Figure S1: Neonatal Se deficient mice demonstrate alveolar development at P7 with no sex differences; Figure S2: Neonatal Se deficient mice demonstrate decreased activity of GPx and Txnrd at postnatal day 0 and 7, with no differences by sex; Figure S3: Se deficient neonates demonstrate decreased pulmonary selenoprotein N content at P0 and P7, without differences by sex.
