# Supplementary material for: Neonatal Selenium Deficiency Decreases Selenoproteins in the Lung and Impairs Pulmonary Alveolar Development
Source: Antioxidants (Basel). 2022 Dec 7;11(12):2417. doi: 10.3390/antiox11122417 (PMC9774937; doi:10.3390/antiox11122417)
Supplement: Supplementary file 1 [file antioxidants-11-02417-s001.zip › antioxidants-1988205-supplementary.pdf]

Table S1. List of antibodies used for western blot analysis		
Anti-Gpx1	1:500	R&D Systems; Minneapolis, MN
Anti-Gpx3	1:500	R&D Systems; Minneapolis, MN
Anti-Txnrd1	1:2000	TE Tipple lab; Oklahoma City, OK
Anti-Trxrd2	1:1000	Santa Cruz; Santa Cruz, CA
Anti-Selenoprotein N	1:500	Santa Cruz; Santa Cruz, CA
Anti-SOD1	1:1000	Abcam; Cambridge, UK
Anti-SOD2	1:500	Millipore; Billerica, MA
Anti-SOD3	1:200	R&D Systems; Minneapolis, MN
Anti-Catalase	1:1000	Abcam; Cambridge, UK

Table S2. List of genes and primers used for qPCR analysis	
Gpx1	Mm04207457_g1
Gpx3	Mm00492427_m1
Txnrd1	Mm00443675_m1
Trxrd2	Mm00496766_m1
Nqo1	Mm01253561_m1
Gclc	Mm00802655_m1
Hmox1	Mm00516005_m1
Selenon	Mm01188435_m1
18S	Mm03928990_g1

Figure S1

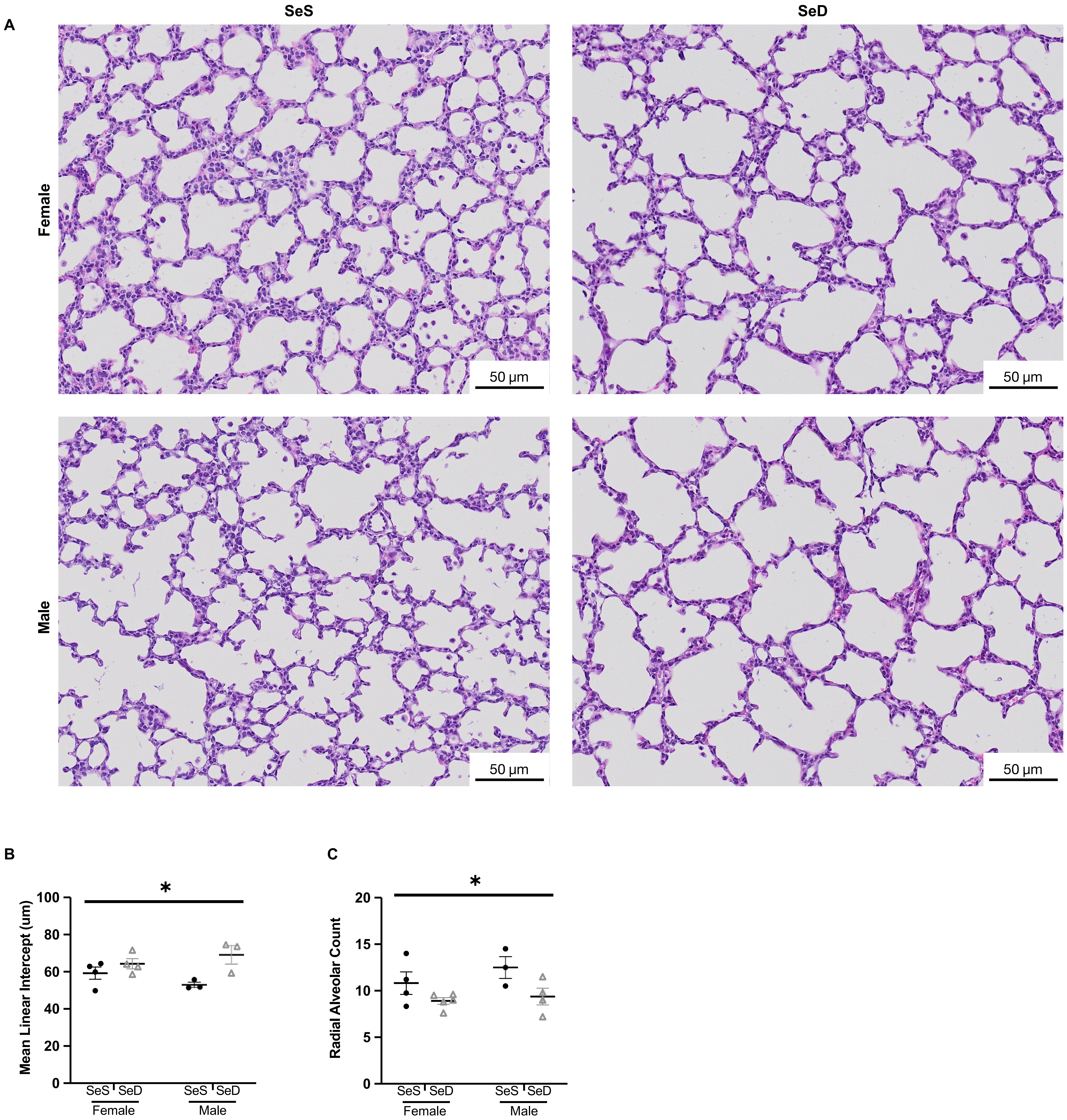

Figure S1. Neonatal Se deficient mice demonstrate impaired alveolar development at postnatal day 7, with no sex differences. C57Bl/6 mice were placed on diets that differed only in Se content, either 0.4 ppm or <0.01 ppm of sodium selenite, through pregnancy and lactation. Alveolar development was assessed at postnatal day 7. Analysis was done by sex. (A) Representative images, (B)Mean linear intercept, (C) Radial alveolar counts. N = 3-4 for all groups. Data are presented as mean (±SEM), Two-way Anova with Tukey's correction for multiple comparisons. *$p < 0.05$ for diet only.

Figure S2

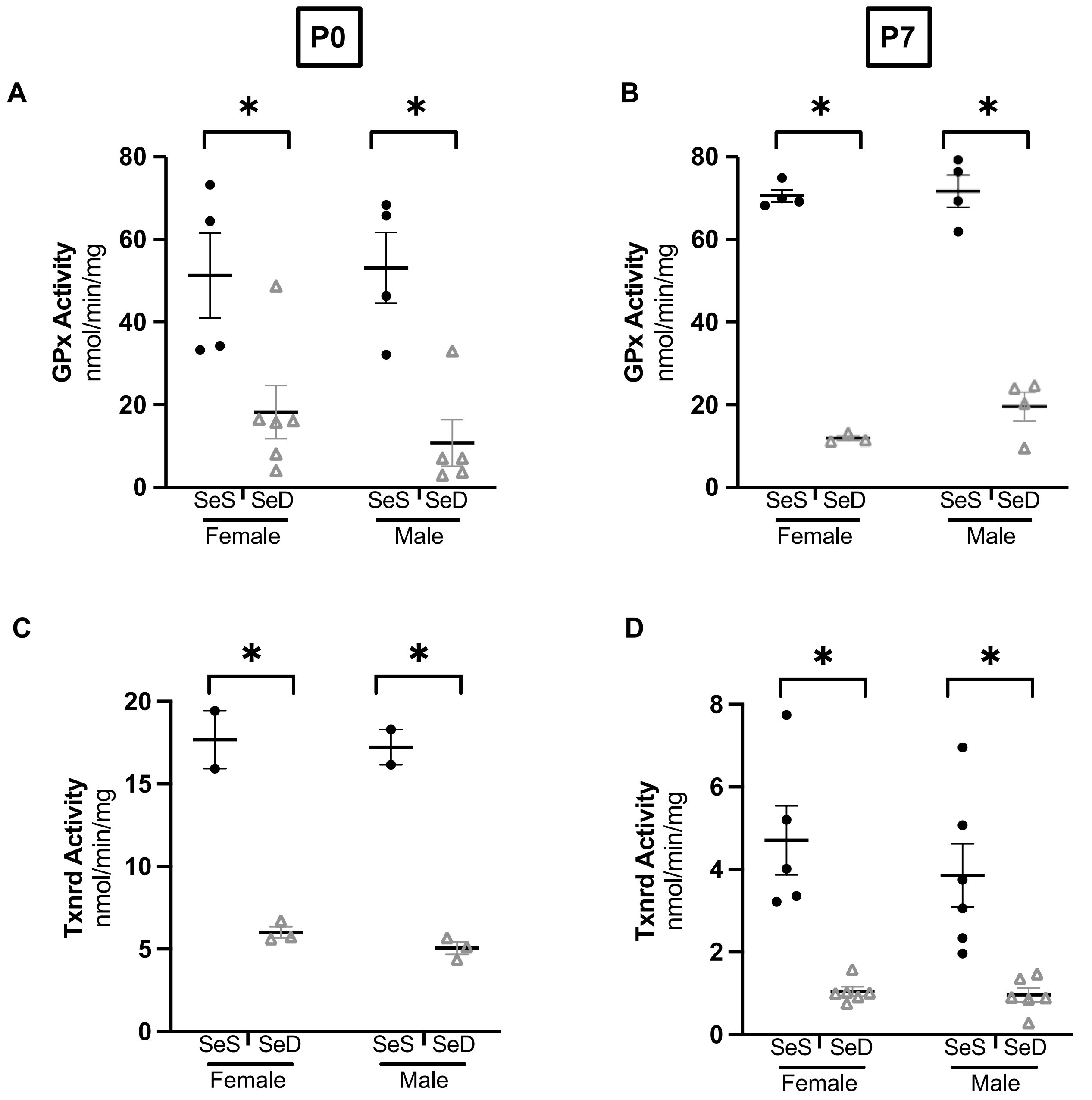

Figure S2. Neonatal Se deficient mice demonstrate decreased activity of GPx and Txnrd at postnatal day 0 and 7, with no differences by sex. C57Bl/6 mice were placed on diets that differed only in Se content, either 0.4 ppm or <0.01 ppm of sodium selenite, through pregnancy and lactation. Pulmonary organ homogenate was evaluated on day of birth (P0) and postnatal day 7 (P7). Analysis was done by sex. (A) Glutathione peroxidase activity levels at P0, (nmol/minute/mg of protein) (B) Glutathione peroxidase activity level at P7, (nmol/minute/mg of protein). (C) Thioredoxin reductase activity levels at P0, (nmol/minute/mg of protein). (D) Thioredoxin reductase activity level at P7, (nmol/minute/mg of protein). N=2-6. Data are presented as mean (±SEM), Two-way Anova with Tukey's correction for multiple comparisons. *p< 0.05 by multiple comparisons.

Figure S3

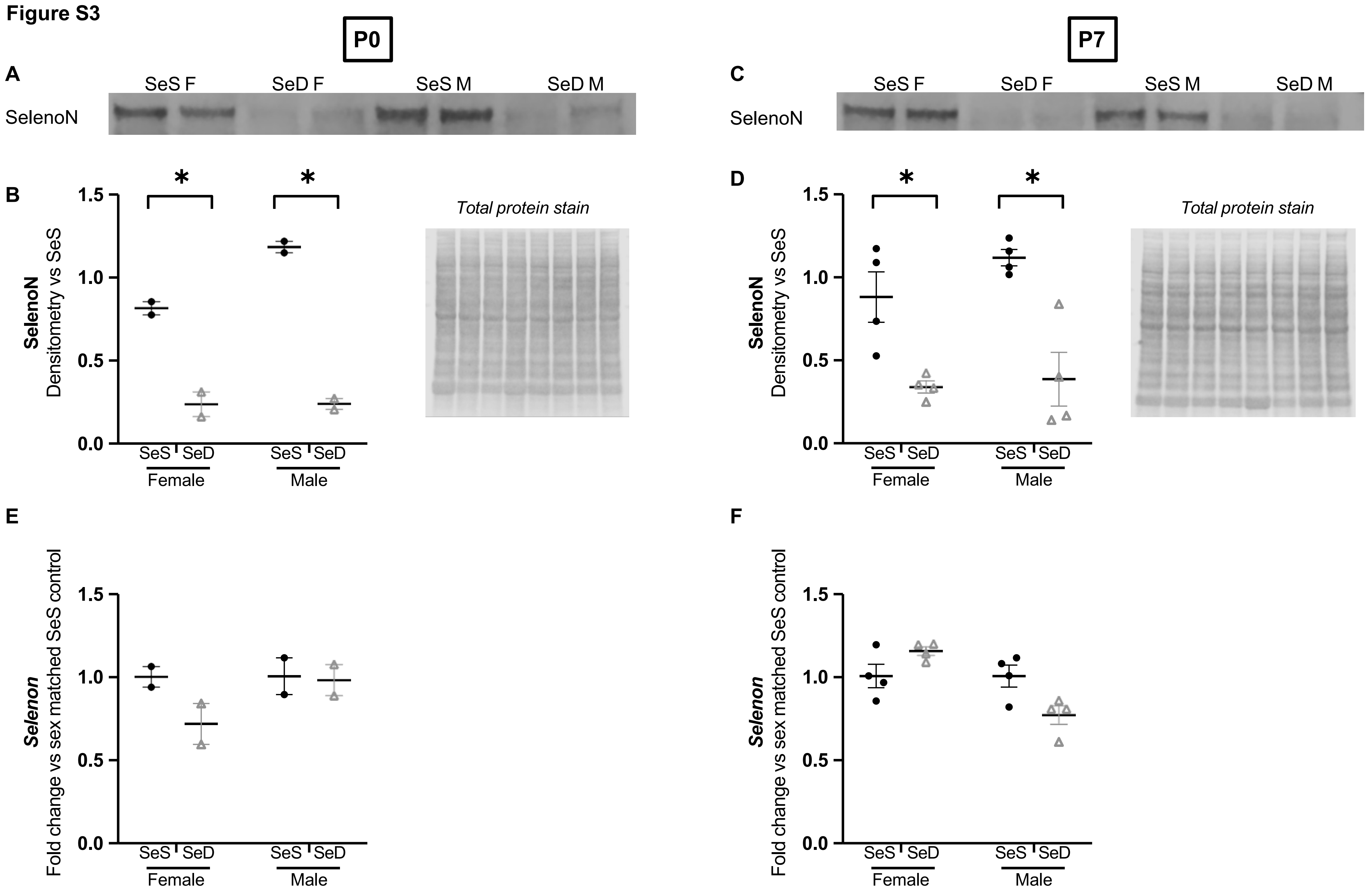

Figure S3. Se deficient neonates demonstrate decreased pulmonary selenoprotein N content at P0 and P7, without differences by sex. C57Bl/6 mice were placed on diets that differed only in Se content, either 0.4 ppm or <0.01 ppm of sodium selenite, through pregnancy and lactation. Pulmonary organ homogenate was evaluated for the offspring on day of birth (P0) and postnatal day 7 (P7). Analysis was done by sex. (A) Representative Western blots of pulmonary selenoprotein N for SeS P0 samples and SeD P0 samples, Densitometric analysis of (B) selenoprotein N protein content expression at P0. Results are normalized to total protein stain and expressed as a ratio to SeS mice. (C) Fold change in *Selenon* mRNA at P0. (D) Representative Western blots of pulmonary selenoprotein N for SeS P7 samples and SeD P7 samples, Densitometric analysis of (E) selenoprotein N protein content expression at P7. Results are normalized to total protein stain and expressed as a ratio to SeS mice. (F) Fold change in *Selenon* mRNA at P0. N=2-4. Data are presented as mean (±SEM), Two-way Anova with Tukey's correction for multiple comparisons. *p< 0.05 by multiple comparisons testing
